# Liver Impairment—The Potential Application of Volatile Organic Compounds in Hepatology

**DOI:** 10.3390/metabo11090618

**Published:** 2021-09-11

**Authors:** Georgios Stavropoulos, Kim van Munster, Giuseppe Ferrandino, Marius Sauca, Cyriel Ponsioen, Frederik-Jan van Schooten, Agnieszka Smolinska

**Affiliations:** 1 Department of Pharmacology and Toxicology, NUTRIM School of Nutrition and Translational Research, Maastricht University, 6227 AP Maastricht, The Netherlands; g.stavropoulos@maastrichtuniversity.nl (G.S.); marius.sauca@outlook.com (M.S.); f.vanschooten@maastrichtuniversity.nl (F.-J.v.S.); 2 Department of Gastroenterology and Hepatology, Amsterdam University Medical Centres, Academic Medical Center, 1000 AE Amsterdam, The Netherlands; k.n.vanmunster@amsterdamumc.nl (K.v.M.); c.y.ponsioen@amsterdamumc.nl (C.P.); 3 Owlstone Medical, Cambridge CB4 0GB, UK; giuseppe.ferrandino@owlstone.co.uk

**Keywords:** VOCs, liver diseases, breath, faeces, bile, urine, noninvasive

## Abstract

Liver diseases are currently diagnosed through liver biopsy. Its invasiveness, costs, and relatively low diagnostic accuracy require new techniques to be sought. Analysis of volatile organic compounds (VOCs) in human bio-matrices has received a lot of attention. It is known that a musty odour characterises liver impairment, resulting in the elucidation of volatile chemicals in the breath and other body fluids such as urine and stool, which may serve as biomarkers of a disease. Aims: This study aims to review all the studies found in the literature regarding VOCs in liver diseases, and to summarise all the identified compounds that could be used as diagnostic or prognostic biomarkers. The literature search was conducted on ScienceDirect and PubMed, and each eligible publication was qualitatively assessed by two independent evaluators using the SANRA critical appraisal tool. Results: In the search, 58 publications were found, and 28 were kept for inclusion: 23 were about VOCs in the breath, one in the bile, three in urine, and one in faeces. Each publication was graded from zero to ten. A graphical summary of the metabolic pathways showcasing the known liver disease-related VOCs and suggestions on how VOC analysis on liver impairment could be applied in clinical practice are given.

## 1. Introduction

Fetor hepaticus, a musty breath aroma, has been among the most prominent liver insufficiency signs available to clinicians, and it was in the 1970s when Chen et al. [1] identified the first responsible compounds. The authors reported that several mercaptans and aliphatic acids (i.e., predominantly acetic and propionic acid) were elevated in the exhaled breath of individuals with liver cirrhosis [2]. However, it was not until the 1990s that Tangerman et al. [3] pinpointed dimethyl-sulphide as the primary source of fetor hepaticus. These studies [1,2,3] were the first liver-related volatile organic compound (VOC) analyses in the breath and paved the way for further research in the field. Many pathophysiological conditions in the liver alter various hepatic metabolic pathways, modifying the abundance of specific exhaled VOCs. Derivatives of cell membrane peroxidation can generate different VOCs as a result of oxidative stress in hepatic inflammation. Metabolic pathway alterations can lead to increased amounts of several compounds, such as sulphur derivatives, through the incomplete transamination of sulphur-containing amino acids [1] or ammonia through the altered urea cycle [4]. Elevated ketones can result from a combination of impaired hepatic gluconeogenesis, increased insulin resistance, and glycogen exhaustion [5], whereas exhaled acetic and propionic acid increase due to reduced hepatic clearance of short-chain fatty acids from the gut microbiome as a result of increased sinusoidal pressure and portosystemic shunts [1]. Many liver diseases that ensue in the sequence of hepatitis, fibrosis, cirrhosis, and end-stage liver failure still pose diagnostic and monitoring challenges: non-alcoholic fatty liver disease (NAFLD), non-alcoholic steatohepatitis (NASH), autoimmune hepatitis (AH), chronic cholestatic diseases including primary sclerosing cholangitis (PSC), and primary biliary cirrhosis are such examples. All these conditions require an invasive liver biopsy for diagnosis, which frequently does not confirm but rather suggests a specific diagnosis. Metabolically, the liver is the main active organ; therefore, VOC analysis in the breath and other body fluids or faeces could hold great noninvasive, patient-friendly potential for diagnostic purposes and for gauging functional reserve of liver impairment.

### 1.1. Liver Pathophysiology and Liver Function Tests

A wide variety of viral, immune-mediated, cholestatic, and toxic conditions may cause chronic liver tissue inflammation. In response to this, the liver accumulates extracellular matrix components, leading to fibrous tissue and scarring [6,7]. In prolonged and severe liver damage, fibrosis might turn into cirrhosis and end-stage liver disease. Substantial liver damage leads to impaired liver function, causing health issues such as disturbed coagulation and hepatic encephalopathy. Moreover, increased hepatic flow resistance leads to portal hypertension that causes hemodynamic insufficiency, which subsequently leads to ascites, varices, and several other critical conditions [8]. Finally, liver cirrhosis is a premalignant condition with an increased risk for hepatocellular carcinoma [9]. Diagnosis and monitoring of liver disease progression are essential to establish an optimal treatment strategy and evaluate therapeutic effects [10]. However, only a handful of biomarkers demonstrate sufficient specificity and sensitivity to develop a reliable diagnosis and monitoring of chronic liver injury. For example, anti-mitochondrial antibodies are used to diagnose primary biliary cholangitis, whereas polymerase chain reaction is used for viral hepatitis. However, both approaches fail to indicate the severity of liver injury. Liver biopsy is considered the reference method for diagnosis and evaluation of liver impairment, although its invasiveness and cost make it less suitable for frequent sampling. Additionally, in some liver diseases, such as cholestatic liver diseases, liver fibrosis is patchy and not homogenous, which decreases the representability, and thus, accuracy of the biopsy.

In the past few decades, several noninvasive biomarkers have entered the liver research field, some of which have already been used in clinical trials, and the most widely used are the enhanced liver fibrosis score (ELF) [11], the FibroTest [12], and the Pro-C3 [13]. All these biomarkers measure molecules involved in fibrogenesis or fibrinolysis; however, they are influenced by confounding factors (e.g., fibrous tissue elsewhere), leading to suboptimal sensitivity and specificity [14]. Moreover, liver fibrosis can be detected through imaging techniques such as ultrasound elastography, which measures liver stiffness (liver fibrosis has been associated with liver stiffness) and is currently widely used in clinical trials and daily clinical practices. Other imaging techniques include magnetic resonance imaging (MRI), computed tomography (CT), or magnetic resonance elastography. However, other pathophysiological processes that increase liver stiffness, such as cholestasis, decrease elastography reliability in its capability to measure fibrosis [14]. Concerning the liver functional reserve, which is vital to determine the moment patients qualify for liver transplantation, the end-stage liver disease model (MELD) is widely applied [15]. This model uses serum bilirubin, the international normalised ratio (INR) for prothrombin time (i.e., a measure of clotting factors), and serum creatinine; these parameters combined to constitute a model as a proxy for the liver function that predicts mortality within 90 days. Mortality and disease severity should be considered; however, the combination of such parameters makes the model dependent on a kidney function read-out, which is not an optimal solution either [16]. Despite the different invasive and noninvasive methods to assess liver diseases, more than 50% of the cases are detected at advanced stages when decompensation episodes occur [8,17]. As a result, the need for new, reliable, and effective biomarkers in the context of liver function or disease diagnosis remains.

Breath tests are already used in clinical setups; an example is identifying *Helicobacter pylori* infection via the C13 urea breath test [18]. Here, labelled C13 urea is administered to patients, and then their exhaled breath is collected, where the isotope-labelled carbon dioxide is measured. Other C13 breath tests, such as the C13 aminopyrine breath test, have also been used to examine liver diseases [19,20]; however, C13 implementations are outside the scope of the present review since they are not based on VOC analysis. The current review focuses on endogenously formed compounds that have been connected with liver impairment, among which are nitrogen derivates [4], ketones [21], alkanes [21], sulphur derivates [1], and alcohols [22].

### 1.2. VOC Analysis

In human research, VOCs arise from different body matrices such as breath, faeces, urine, bile, breast milk, and blood, resulting from exogenous or endogenous sources [23,24,25]. Exogenous VOCs originate from the gut microbiome or the environment. The latter are absorbed through the skin, inhaled, or ingested with food and beverages. Moreover, they might be the result of therapeutic interventions [26]. A compound is considered endogenous when its concentration in a subject/patient sample is higher than in ambient air [27]. Endogenous VOCs are produced biochemically by body cells and tissues, such as lung and airway tissues, or from other organ tissues (e.g., liver or kidney) [28]; these VOCs are a reflection of biochemical reactions such as apoptosis, inflammation or oxidative stress [29,30,31]. These VOCs arise from body chemical reaction cascades in diseased individuals due to cellular damage [32]; they are released in the bloodstream and spread among the body excretions. In particular, liver diseases alter VOC abundances in the blood [33,34], leading to different amounts of VOCs present in body excretions.

Many studies have explored different approaches to quantifiably detect VOCs in liver disease patients [22,33,34,35]. The vast majority of these studies examined breath as the means of discovering discriminatory VOCs, whereas only a handful of studies used body excretions other than breath [24,36,37]. Thus far, examining liver diseases via VOC analysis has mainly focused on cirrhosis and NAFLD, and currently, no VOC detection test has been implemented in the clinics yet, despite the diagnostic potential of VOC analysis in general [38,39,40]. This review aims to discuss the available VOCs literature on liver diseases examined through, primarily, breath, and secondarily, through faeces, urine, and bile. Finally, conclusions on possible causes for the lack of clinical VOC tests for liver diseases are drawn, and possible future directions are suggested.

## 2. Materials & Methods

### 2.1. Literature Search

The scientific literature search focused on liver disease diagnosis, prognosis, and monitoring via VOCs in the breath or faeces. For breath-related VOCs, PubMed and ScienceDirect were interrogated with the following search terms:

(((((liver disease) OR “Liver Diseases”[Mesh]) OR ((Diagnosis/Broad[filter]) AND (“Liver Diseases”[Mesh])))) AND ((volatile organic compounds) OR “Volatile Organic Compounds”[Mesh])) AND ((breath analysis) OR “Breath Tests” [Mesh]).

The search terms for faeces were:

(((((“Liver Diseases”[Mesh]) OR liver disease) OR ((Diagnosis/Broad[filter]) AND (“Liver Diseases”[Mesh])))) AND ((volatile organic compounds) OR “Volatile Organic Compounds”[Mesh])) AND (((fecal analysis) OR faecal analysis) OR “Feces” [Mesh]).

**Figure 1:**
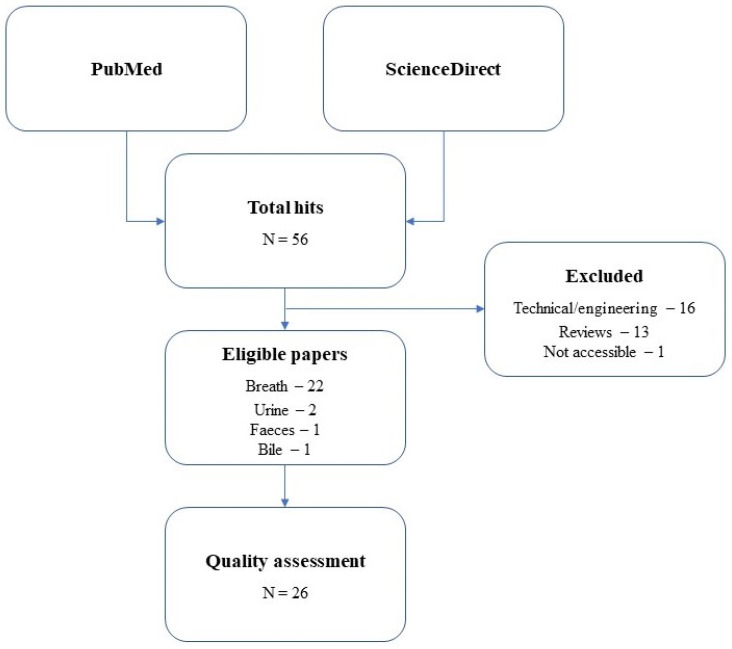
Schematic representation of the literature search performed in the present review. The total number of papers found is 58, and the number of publications eligible to be reviewed is 28.

Replacing the word “Diagnosis” with “Prognosis” or “Monitoring” yielded the same results for both biological matrices. Additional studies cited by the initially identified research papers were also included and discussed in this review. These additional studies examined liver diseases related to VOCs in the breath and faeces and other body fluids such as urine, blood, and bile. The number of the latter was minimal; therefore, it was decided to discuss these as well. Only articles published in English, reporting original research in humans, and focused on different VOC patterns between healthy and diseased liver subjects were included. Engineering or technical studies were excluded since they fall outside the scope of this review. Finally, no year of publication criterion was imposed as an exclusion criterion. An overview of the literature search and the exact numbers of the publications found and used herein can be seen in the Results section in Figure 1.

### 2.2. Quality Assessment

Two independent evaluators assessed the eligible studies using the Scale for the Assessment of Narrative Review Articles (SANRA) [41]. SANRA is a brief critical appraisal tool used to assess the quality of narrative reviews and research articles, and it consists of a six-question questionnaire. Each question is evaluated on a scale from zero to two (i.e., 0, 1, and 2), resulting in a maximum cumulative score of 12 for the paper at hand. However, in the present review, question number three (“Description of literature search”) was excluded from the evaluation of the papers because it is not applicable for scientific research papers. The whole SANRA questionnaire can be found elsewhere [41]. As a result, the SANRA assessment score was on a scale from zero to ten. Papers with a maximum aggregate score of five (i.e., (0–5]) were considered as low-quality, those with a total score from five to seven (i.e., (5–7]) were regarded as a medium-quality, and those with an aggregate score from seven to ten (i.e., (7–10]) were considered as high-quality. However, the SANRA quality assessment tool was deemed not strict enough when the assessment was finalised (i.e., almost all the papers were scored with eight or more; the scores are illustrated in the Results Section, in Table 1). This is because the questions are made to assess general scientific guidelines; thus, five additional assessment questions were included in the overall assessment. The two assessors construed these questions following the present review purposes; these questions can be seen in the Supporting Information in Table S1. The new questions were also graded on a scale from zero to two (the same as the SANRA questions), and the new scores (i.e., from the five SANRA questions and the added five summed up) are also illustrated in Table 1.

## 3. Results

The literature search performed in both PubMed and ScienceDirect resulted in 58 hits in total, of which 1 was not accessible, 16 were either engineering or technical, and 13 were reviews. Thus, the final number of papers to be discussed here was 28. From these 28 articles, 23 found VOCs in the breath, one in the bile, three in urine, and one in faeces.

Table 2 summarises all the compounds that were found as significant in more than one of the examined research papers analysed in the present review. Table 2 also describes what is believed to be the biological origin of each of the present compounds.

## 4. Discussion

### 4.1. Differentiation among General Cirrhotic CLD, Non-Cirrhotic CLD, and Healthy Individuals

Pauling et al. pioneered breath testing with their unprecedented study published in 1971 [64]. Since then, the 500+ discovered VOCs have provided insights into the human body metabolic processes. Lipid peroxidation has been associated with alkanes such as pentane and ethane, whereas cholesterol metabolism has been linked to isoprene and other unsaturated compounds [27,28,52,65]. Dextrose metabolism has been correlated with ketones such as acetone, while the sulphur-containing compounds dimethyl-sulphide, methyl-mercaptans, and ethyl-mercaptans, have been associated with renal failure or liver disease and deemed the cause of fetor hepaticus of cirrhotic patients [27,28,52,65]. Initial studies mainly focused on finding biomarkers related to liver cirrhosis. Hiroshi et al. [43], Tangerman et al. [66], and Friedman et al. [42] paved the way for modern liver breath analysis by comparing cirrhotic patients to healthy controls, aiming to identify compounds that differ between the two cohorts by exploiting advances of the gas chromatography-mass spectrometry (GC-MS) technology. All three studies found significantly higher levels of dimethyl-sulphide in the breath of cirrhotic patients. However, Friedman et al. [42] also reported that hydrogen-sulphide was substantially higher in patients with less severe forms of cirrhosis than healthy controls. More interestingly, they also found elevated levels of limonene in half of the cirrhotic patients. The additionally reported compounds in the [42] study might have resulted from the fact that the GC detector used was different than the one used in the [43,66] studies.

Van den Velde et al. [33] and Dadamio et al. [45] also analysed liver cirrhosis patients’ and healthy controls’ breath to identify VOCs related to liver cirrhosis by using GC-MS. Van den Velde et al. found that acetone, dimethyl-sulphide, 2-butanone, and 2-pentanone were elevated, while indole and dimethyl-selenide were reduced in the patients compared to controls. The discriminative model based on these compounds showed a sensitivity and specificity of 100% and 70%, respectively. Dadamio et al. found more than 20 compounds elevated in the breath of cirrhotic patients. The resulting classification models provided an overall average sensitivity and specificity of 83% and 100%, respectively. Morisco et al. [22] also stratified cirrhotic patients and healthy volunteers to evaluate the capability of breath testing in distinguishing among different levels of disease severity in addition to liver cirrhosis diagnosis, employing proton transfer reaction-MS (PTR-MS). Twelve compounds (i.e., heptadienol, methanol, 2-butanone, 3-pentone, 2-octanone, C8-ketone, 2-nonanone, C9-ketone, monoterpene, p-cymene, sulphoxide compounds, an S-compound, an NS-compound, and an N-compound) had significantly higher concentrations, except for the S-compound, which had significantly lower concentration, in liver cirrhosis patients compared to controls. Morisco et al. [22] further stratified their patients into two groups (i.e., mild cases and severe cases) to assess the different VOC concentrations according to disease severity. They found that five VOCs (i.e., heptadienol, C8-ketone, monoterpene (tentatively identified as limonene), 2-butanone, and an NS-compound) had higher concentrations in the severe cases, while the S-compound and the N-compound had lower concentrations in the severe cases. Limonene had the highest diagnostic performance with a sensitivity and specificity of 83% and 86%, respectively. Mild cases were discriminated from controls with a sensitivity and specificity of 83% and 86%, respectively, and with a sensitivity and specificity of 100% from the severe cases. Interestingly, the monoterpene, tentatively identified as limonene, had the highest diagnostic performance again with a sensitivity and specificity of 100% when discriminating mild from severe cases. In general, the [22] study found different compounds than the [33,45] studies (Table 3); however, the chemical classes of the discovered VOCs were the same (i.e., sulphur compounds and ketones). PTR-MS seems to provide a more complex picture of the breath compounds in liver cirrhosis patients and it seems to be able to distinguish between different disease severity classes, which may explain the identification of different compounds in the [22] study. Of note is that the [33,45] studies did not enforce a fasting state for their volunteers, whilst the [22] study did, and fasting could explain the appearance of ketone bodies in the breath.

In 2015, Del Rio et al. [47] also compared cirrhotic patients against healthy cohorts and aimed to identify breath biomarkers of liver diseases by employing PTR-MS. Cirrhotic patients who had undergone a liver transplant were compared to their pre-transplant samples, effectively becoming their controls and allowing liver metabolism-related compound isolation. It was found that methanol, 2-butanone, carbon disulphide, 2-pentanone, and limonene presented significantly higher concentrations in liver cirrhosis patients than in controls (Table 3). Limonene levels were monitored in post-liver transplant patients, and they were steadily decreasing in the following days. Results generated by this study design support the claim of Del Rio et al. that all previous studies were only hypothesis-generating, since there was a lack of follow-up to confirm the found biomarkers. These findings also highlight limonene potential as a liver function biomarker in liver transplant patients by monitoring its wash-out [47]. It should be noted, however, that post-liver transplantation and other factors could have influenced the limonene levels, such as reduced food intake in the first days after the operation.

Pijls et al. [46] stratified CLD patients with or without cirrhosis and aimed to identify a VOC profile to separate the classes using GC-MS. They identified 11 VOCs (i.e., dimethyl-sulphide, terpene (limonene), 2-methybutanal, propanoic acid, octane, terpenoid, 3-carene, 1-hexadecanol, an unknown compound, as well as a branched C16H34) that discriminated between non-cirrhotic CLD and cirrhotic CLD patients with an accuracy of 84.1% (Table 3).

De Vincentis et al. [50] also compared cirrhotic against non-cirrhotic patients and healthy controls using the emerging e-nose technology, which provides rapid breath-prints (BPs). This technique offers a VOC profile on a point-of-care base because it can be performed instantaneously in an outpatient care setting. De Vincentis et al. identified BPs that discriminate different liver disease severity stages among liver cirrhosis patients with a sensitivity and specificity of 87.5% and 64.7%, respectively. Differences among patients with infectious and non-infectious liver diseases were also achieved with a sensitivity and specificity of 29% and 88%, respectively (Table 3). It is worth mentioning that in a follow-up study, De Vincentis et al. [67] showed that e-nose could significantly identify cirrhotic patients with a high risk of hospitalisation and mortality, thus making it a substantial alternative to the Child–Pugh and MELD scores in clinical practices, which are considered as the reference method. Successful e-nose discriminatory capabilities have been reported already [68,69].

In 2015, Eng et al. [48] conducted the first reported paediatric study to differentiate cirrhotic children from healthy children by using the newly developed selected ion flow-tube-MS (SIFT-MS). They identified 1-decene, 1-heptene, 1-octene, and 3-methyl-hexane as significantly higher in cirrhotic children than in controls. These VOCs were also increased in children with advanced liver fibrosis compared to children suffering from no to mild fibrosis. Additionally, 1-nonene, (E)-2-nonene, and dimethyl-sulphide were lower in cirrhotic children than controls and inversely proportional to the degree of liver fibrosis. This finding is unexpected and contradicts previous studies conducted in adults [22,33,45,47], where dimethyl-sulphide was elevated in adult liver disease patients. However, this inconsistency may be explained by differences in hepatic metabolism between children and adults [70]. Eng et al. also generated a predictive model by combining five VOCs (i.e., 1-octene, triethyl-amine, ethane, E2-nonene, and 1-decene) that showed prediction accuracy of cirrhosis with an AUC of 0.97 (Table 3).

#### 4.1.1. Origin of the VOCs Reported in General Cirrhotic CLD against Healthy Individuals

**Figure 2:**
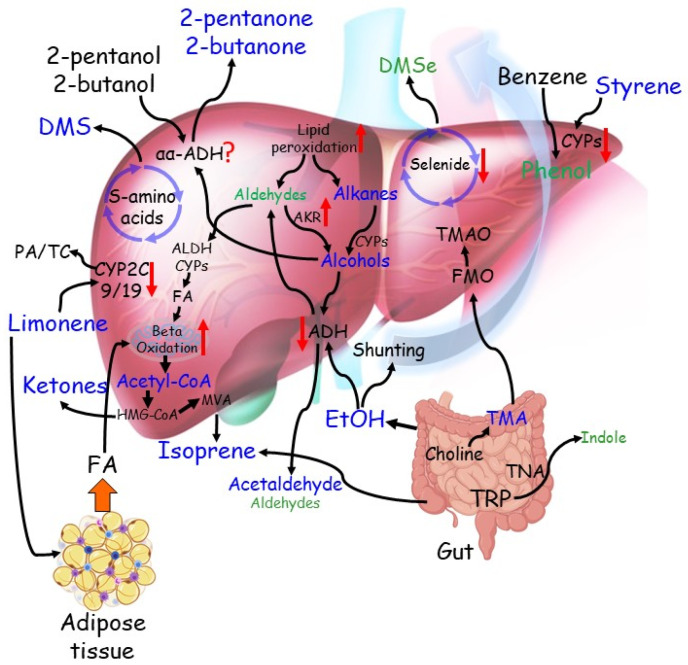
The complex network of established and proposed metabolic pathways from which VOCs stem and their alterations in chronic liver diseases. Compounds found elevated in the breath of patients with cirrhosis are indicated in blue, those downregulated in green. Red arrows indicate changes in the metabolic pathways. From the bottom left: insulin resistance increases fatty acid (FA) shuttling from the adipose tissue to the liver. The resulting excess of acetyl-CoA is metabolised in the mevalonate pathway (MVA) to ketones and isoprene, the latter also generates from gut microbiota. Dietary limonene is converted to Perillyl alcohol (PA) and trans-Carveol (TC) mainly by CYP2C9 and CYP2C19. PA and TC are more soluble in the aqueous environment and can be excreted in urine. In the cirrhotic liver, reduced activity of CYP enzymes leads to the accumulation of limonene in the adipose tissue and increases its permanence in the body, resulting in elevated levels in the breath. Incomplete metabolism of sulphur-containing amino acids in the transamination pathway, coupled with Cytochrome C oxidase deficiency in the cirrhotic liver, lead to elevated levels of Dimethyl-sulphide (DMS) in the breath of patients with cirrhosis. Dietary 2-butanol, a flavouring agent, and a compound contained in fruit is converted to 2-butanone by αα-ADH. A similar pathway may also involve 2-pentanol, a similar compound. Both 2-butanone and 2-pentanone have been found elevated in the breath of patients with cirrhosis. Lipid peroxidation, a process triggered by increased inflammation of the cirrhotic liver, has been proposed to generate alkanes, such as octane, pentane and ethane, and medium, long-chain aldehydes. These alkanes have been found elevated, while detected aldehydes are reduced. Both classes of compounds can be converted to corresponding alcohols by CYPs or aldo-keto reductases (AKR), respectively. Medium-chain primary alcohols can be further metabolised by alcohol dehydrogenases (ADH) back to aldehydes, which can be converted to corresponding fatty acids and feed beta-oxidation. Secondary alcohols such as 2-butanol and 2-butanone may also be generated and contribute to increasing the corresponding ketones. Ethanol (ETOH), which originates from the diet, sugar fermentation from gut microbiota, and oxidation of ethane, increases in the breath of patients with cirrhosis because of shunting and downregulation of the main metabolising pathway. However, acetaldehyde, the main bio-product of ETOH metabolism, has also been elevated due to downregulation of the downstream enzyme aldehyde dehydrogenase (ALDH). Dimethyl selenide (DMSe) is one of the excretion products of selenide metabolism. Selenide blood levels were reduced in patients with cirrhosis, to an extent related to disease severity. Therefore, reduced DMSe in breath may result from a lack of substrate and impaired selenide metabolic pathway. Benzene is a pollutant generated mainly by petrol products and readily adsorbed by the body by inhalation. Benzene is oxidised to phenol by the CYP system. Reduced CYP activity in cirrhosis may explain reduced breath levels of phenol. Exposure to styrene takes place mainly by adsorption of vapours through the lungs. Its reduced oxidation by the CYP system explains its increase in the breath of patients with cirrhosis. Trimethylamine (TMA) is derived from the diet by microbial degradation of precursors such as choline. TMA is readily absorbed and metabolised by flavin-containing monooxygenases (FMO) in trimethylamine N-oxide (TMAO) for urine excretion. Reduced FMO activity in cirrhosis may lead to increased TMA in the breath. Indole is a catabolic product of tryptophane (TRP) metabolism by tryptophanase (TNA) activity of gut microbiota, which alterations in cirrhosis may lead to reduced indole exhalation in the breath.

The most significant compounds, and the ones that the aforementioned literature (Section 4.1) seems to be more certain about their origin, are limonene and dimethyl-sulphide. Limonene is suggested to originate from foods and drinks. Limonene is broken down in the liver by CYP2C19 and CYP2C9 enzymes into other compounds such as perillyl alcohol, trans-isopiperitenol, and trans-carveol [71]. In liver impairment, the CYP2C19 and CYP2C9 enzymes are proportionally reduced and thus leads to increased limonene levels in the body [22,42,47]. Increased dimethyl-sulphide, along with other sulphur-containing compounds, points toward incomplete metabolism of sulphur-containing amino acids in the transamination pathway due to liver impairment. As far as other groups of compounds are concerned, the aforementioned literature also discusses possible metabolic pathways that might be involved in their origin, and they can be summarised as follows. It is suggested that free fatty acids, triglycerides, and ketones such as 2-butanone, 2-pentanone, and acetone may increase due to hepatic insulin resistance [22,33], which favours lipolysis and free fatty acid beta-oxidation. As for reduced indole and phenol levels, they may have resulted from the impaired ability of the liver to degrade aromatic amino acids such as tryptophan [22,33], whereas the reduced dimethyl-selenide is explained by lower levels of this micronutrient observed in the blood of patients with cirrhosis [72]. Increased levels of hydrocarbons, such as ethane and pentane, were attributed to the impaired conversion of saturated hydrocarbons into alcohols due to deficient cytochrome P450 activity [33,45]. Cirrhotic liver inability to metabolise methanol by efficiently using alcohol dehydrogenase [47] or an imbalance in the bacterial flora composition [22] explain the increased methanol levels in liver disease patients, which alters the colon fermentation processes. Finally, high levels of other alkanes such as 3-methyl-trexane, 1-decene, 1-heptene, and 1-octene are thought to be related to oxidative stress [48]. Figure 2 illustrates these suggested pathways.

### 4.2. Differentiation among Specific Cirrhotic CLD, Non-Cirrhotic CLD, and Pre-Cirrhotic CLD

#### 4.2.1. VOCs in Advanced versus Mild Fibrosis Patients

In 2013, Alkhouri et al. [49] assessed the utility of breath VOC measurements to diagnose advanced fibrosis in CLD patients by employing SIFT-MS. They found reduced acetone, benzene, carbon disulphide, isoprene, pentane, and ethane in the breath of patients with advanced fibrosis compared to those with minimal fibrosis (Table 4). Isoprene had the highest AUC for advanced fibrosis (i.e., AUC = 0.855), and 75% of the patients were correctly classified as advanced fibrosis using certain cut-off levels for isoprene.

#### 4.2.2. VOCs in Cirrhotic Patients with Hepatic Encephalopathy or Hepatocellular Cancer

Hepatic encephalopathy (HE) was investigated by Khalid et al. [51]. They sampled alcoholic cirrhotic patients, of which some had HE and some others did not have HE, along with a few non-alcoholic cirrhotic patients, harmful drinkers, and healthy volunteers; ultimately, they aimed to differentiate cirrhotic HE patients from cirrhotic patients without HE or harmful drinkers by using GC-MS. They reported that methyl-vinyl ketone and, likely, isothiocyanato-cyclohexane contributed to the group separation of alcoholic cirrhotic patients with HE and without HE. The model yielded a 90% sensitivity and 87% specificity. Undecane and an unknown compound contributed to the separation of alcoholic and non-alcoholic cirrhotic patients without HE, and the model yielded 78% sensitivity and 69% specificity. 1-methyl-4-(1-methyl-ethenyl)-benzene (p-cymenene) and two unknown compounds contributed to the group separation of alcoholic cirrhotic patients and harmful drinkers without cirrhosis, and the model yielded 88% sensitivity and 85% specificity. Octanal, a compound tentatively identified as 2,6-dimethyl-7-octen-2-ol, and an unknown compound contributed to distinguishing harmful drinkers from healthy volunteers, and the model yielded 71% sensitivity and 93% specificity. Methyl-vinyl ketone and an unknown compound allowed for the discrimination of non-alcoholic cirrhotic patients from healthy controls, and the model yielded 92% sensitivity and 100% specificity. Finally, heptane, 1-methyl-2-(1-methyl-ethyl)-benzene, phellandrene, and 2-methyl-hexane contributed to discriminating the alcoholic cirrhotic group from the healthy volunteers, and the model yielded 97% sensitivity and 93% specificity.

In 2016, O’Hara et al. [52], a follow-up of the [47] study, stratified the population of cirrhosis patients based on the presence of HE and investigated variations in limonene, methanol, and 2-pentane by using PTR-MS measurements. They found that limonene was higher in the breath of patients with HE and was the only compound able to discriminate from non-HE patients. In contrast, 2-penatanone could not discriminate against cirrhotic patients stratified by the presence/absence of HE complication. However, they did not provide sensitivity and specificity results.

Qin et al. [56] compared healthy volunteers, cirrhotic patients without hepatocellular cancer (HCC), and non-cirrhotic patients with HCC to find breath biomarkers that could be used to diagnose HCC patients—they ran a GC-MS/solid-phase micro-extraction analysis (SPME). 3-hydroxy-2-butanone, styrene, and decane appeared the most promising breath biomarkers for HCC patients. 3-hydroxy-2-butanone was the only one that was significantly different among all three groups, and it could discriminate between healthy volunteers and HCC groups with a sensitivity and specificity of 83.3% and 91.7%, respectively. In contrast, the diagnostic accuracy between HCC and cirrhosis groups was lower, with a sensitivity and specificity of 70% and 70.4%, respectively (Table 4). Styrene was not significantly different between the healthy volunteers and HCC groups, while decane was not significantly different between the cirrhosis and HCC groups. These compounds were significantly higher in HCC patients than in healthy volunteers, which suggests that these VOCs result from cancer metabolism, and thus, they may serve as breath biomarkers of HCC. The [52] study also examined VOCs in HCC patients; however, its results are different from those in [56]. The former study only found that HCC patients had significantly lower limonene levels than patients without HCC. These differences might be because the [52] study used PTR-MS instead of GC-MS/SPME that the [56] study used.

Ferrandino et al. [58] followed up on the limonene-related hypothesis and by sampling cirrhotic patients, cirrhotic patients with HCC, and healthy controls, they focused on comparing the exhaled limonene levels of their groups to see how they relate with each other by performing a GS-MS analysis. They reported that limonene concentration was significantly higher in cirrhotic and cirrhotic patients with HCC when compared to healthy individuals. However, no significant differences in limonene levels were found between the two diseased groups. They also reported that limonene levels correlate with serum bilirubin but not with alanine transferase. Consequently, Ferrandino et al. confirmed that breath limonene levels do not change among patients with HCC over underlying cirrhosis from patients with matching cirrhosis severity.

In 2020, another broader scale HCC study was reported by Miller-Atkins et al. [59]. They sampled healthy volunteers, cirrhotic without HCC, non-cirrhotic with HCC, pulmonary hypertension (PA), and colorectal cancer liver disease (CRLD) patients, and they examined specific VOCs reported in the literature to see whether they could achieve separation of their classes and which VOCs are more or less abundant in which group. They ran a SIFT-MS analysis, and they published that pairwise disease comparisons demonstrated that most of the VOCs were present in significantly different relative abundances. Each pairwise disease comparison had several compounds as significant; therefore, only the most significant metabolite associations for each disease are mentioned here. Comparing HCC against healthy volunteers revealed that (E)-2-nonene, ethane, and benzene increased in HCC patients, whereas hydrogen sulphide decreased. Comparing cirrhotic against healthy controls showed that trimethyl-amine and propanol significantly increased in cirrhotic individuals. Furthermore, (E)-2-nonene, acetaldehyde, and ethane significantly increased in PA individuals than healthy volunteers, whereas hydrogen sulphide decreased in that pairwise disease comparison. When CRLD patients were compared against healthy controls, (E)-2-nonene, acetaldehyde, and triethyl-amine significantly increased in CRLD individuals, whereas hydrogen sulphide, acetone, and trimethyl-amine decreased. Lastly, Miller-Atkins et al. found that acetone, acetaldehyde, and dimethyl-sulphide were increased in cirrhotic without HCC patients than in non-cirrhotic with HCC patients, while ethanol was increased in the non-cirrhotic HCC patients than the cirrhotic without HCC patients. The authors’ classification results can be seen in Table 4.

Arasaradnam et al. [62] investigated breath VOCs in non-cirrhotic HE patients compared to healthy individuals; however, they used the e-nose technology. They found that the resulting BP could distinguish the two groups with a sensitivity and specificity of 88% and 68%, respectively. The BP could also differentiate between overt and covert HE, however, with a moderate sensitivity and specificity of 79% and 50% (Table 4). E-nose technology does not quantify individual compounds that form the BP; nevertheless, this might not be a considerable bottleneck depending on the application.

#### 4.2.3. VOCs in Non-Alcoholic Fatty Liver Disease versus Non-Alcoholic Steatohepatitis Patients

Breath analysis has also been implemented to examine obesity-related liver diseases. Solga et al. [5] compared NAFLD patients, of which some had NASH, to explore the diagnostic capability of breath biomarkers against a standard blood serum test; they performed a GC analysis. Acetone concentrations in breath were found to be significantly increased in patients with severe steatosis (grade 2 or 3), steatohepatitis, and NASH compared to patients with mild forms of steatosis, or steatohepatitis, and NASH. Breath ethanol was also positively associated with hepatic steatosis severity, as it was higher in the breath of patients with severe steatosis (grade 2 and 3).

In 2013, Verdam et al. [54] investigated NASH. They sampled NASH and non-NASH patients, and they aimed to separate the classes—they performed a GC-MS analysis. They reported that NASH and non-NASH patients could be discriminated by using three compounds: N-tridecane, 3-methyl-butanotrile, and 1-proponol with a sensitivity and specificity of 90% and 69%, respectively [54] (Table 4). Their results, however, are very different from the research conducted in the [5] study. The lack of control and validation in the [5] study might have been a reason for this difference.

Alkhouri et al. [55] examined the usage of exhaled breath analysis as a diagnostic tool in children. They aimed to separate obese children with NAFLD from obese children without NAFLD by performing a SIFT-MS breath analysis. They discovered that various VOCs (i.e., isoprene, acetone, trimethylamine, acetaldehyde, and pentane) could distinguish NAFLD children from those without NAFLD with an AUC of 0.71 (Table 4). The [55] study findings, though, might be questionable since NAFLD was not diagnosed by liver biopsy but by assessing the presence of fatty infiltration.

#### 4.2.4. VOCs in Alcoholic and Non-Alcoholic Fatty Liver Disease Patients versus Cirrhotic Patients

Millonig et al. [35] demonstrated the usage of exhaled breath VOCs for differentiating among non-cirrhotic alcoholic fatty liver disease (AFLD), non-cirrhotic NAFLD, cirrhotic patients, and healthy cohorts. They aimed to separate these groups of patients by using ion-molecule reaction-MS (IMR-MS) analysis. Millonig et al. reported that 19 compounds showed significantly different exhalation patterns (no compound identification was achieved per class) among the different liver disease types. The most promising compound was acetaldehyde, which was significantly higher in NAFLD and AFLD when compared to healthy controls and cirrhotic patients, and ethanol, which was only increased in cirrhotic patients and not in patients with NAFLD, AFLD, or healthy controls (Table 4).

In 2020, Sinha et al. [57] were the latest to investigate the ability to diagnose NAFLD using exhaled breath. They found that styrene, acetone, isoprene, terpinene, dimethyl-sulphide, acetophenone, and limonene significantly differed among cirrhotic and non-cirrhotic NAFLD patients. More specifically, isoprene, acetophenone, and terpinene were significantly lower in non-cirrhotic NAFLD patients than healthy controls; terpinene had the highest predictive capability, achieving an AUC value of 0.84. Styrene, isoprene, acetophenonene, and terpinene were significantly lower in cirrhotic NAFLD patients than healthy controls, whereas dimethyl-sulphide and limonene were significantly higher in cirrhotic NAFLD patients than in healthy controls—limonene and dimethyl-sulphide combined yielded the highest predictive capability with an AUC value of 0.98. Furthermore, dimethyl-sulphide and limonene were significantly higher in cirrhotic NAFLD patients than non-cirrhotic NAFLD; combined, they achieved an AUC of 0.91 (Table 4).

Letteron et al. [44] conducted a large scale study in which they stratified various liver disease patients. They sampled non-alcoholic liver disease patients categorised into acute hepatitis, chronic hepatitis, viral cirrhosis patients, polyadenomatosis of the liver patients, non-alcoholic HCC, liver metastasis, sclerosing cholangitis, biliary cirrhosis, extrahepatic bile duct obstruction patients, alcohol abusers, as well as healthy individuals. They measured the exhaled ethane levels by using a GC-flame ionisation detector (FID). Their results showed that alcohol abusers had significantly higher ethane levels than other non-alcoholic groups.

#### 4.2.5. VOCs in Alcoholic Hepatitis Patients versus Cirrhotic Patients

Hanouneh et al. [21] published a study where they investigated alcoholic hepatitis (AH). More specifically, they gathered two groups that consisted of AH patients with liver cirrhosis, patients with acute decompensation (AD) with aetiologies other than alcohol and liver cirrhosis, and a healthy cohort. They aimed to find concentrations of VOCs that correlate with AH diagnosis and the severity of liver disease in AH patients—patient samples were analysed utilising SIFT-MS. Six compounds were identified to be significantly higher in the exhaled breath of liver disease patients compared to controls: acetaldehyde, 2-propanol, ethanol, acetone, pentane, and trimethyl-amine (TMA). Moreover, four compounds (i.e., acetaldehyde, acetone, TMA, and pentane) stood out in patients with cirrhotic AH compared to patients with AD. Finally, Hanouneh et al. also demonstrated that cirrhotic AH patients have a distinct breath VOC pattern characterised by high levels of acetone, pentane, and TMA when compared against patients with liver disease of aetiologies other than alcohol. Their model created using these three compounds gave an excellent diagnostic accuracy for AH with a 97% sensitivity and a 72% specificity (Table 4).

#### 4.2.6. Origin of the VOCs Reported in Cirrhotic, Non-Cirrhotic, and Pre-Cirrhotic Stage Individuals

The key compounds and their metabolic pathways discussed in the aforementioned literature (Section 4.2.1, Section 4.2.2, Section 4.2.3, Section 4.2.4 and Section 4.2.5) can be summarised as follows. Increased isoprene levels were found in AFLD and advanced fibrosis stage patients [35,49,55], and it is suggested that they are the result of impairment in the cholesterol biosynthesis pathway or that they might be the result of disturbed colon flora. However, other literature suggests that subjects should be at rest before testing because isoprene absence/deficiency maybe the result of exercise and that generally, it should not be attributed to pathophysiological effects onto mevalonate/cholesterol pathways [73,74]. Increased acetone levels were found in stage 1 or 2 fibrosis patients, as well as NAFLD and AH patients [5,21,55]; acetone is believed to be associated with lipolysis and carbohydrate metabolism, where increased expression of the CYP450 enzyme would result in fatty acid beta-oxidation, which then would lead to excess of acetyl-CoA. Another possible explanation could be that reduced NADH levels (Nicotinamide Adenine Dinucleotide) in hepatocellular mitochondria could decrease d-3-hydroxybutyrate and dehydrogenase activity, which also would increase acetone levels. Alkanes such as pentane, heptane, 2-methyl-hexane, and ethane that were found in NAFLD, HE, and AH, and alcohol abusers were linked to lipid peroxidation of polyunsaturated fatty acids due to oxidative stress [21,44,51,55]; terpinene, found in NAFLD individuals, was also linked to oxidative stress [57]. Furthermore, isothiocyanato-cyclohexane was characterised as a common environmental pollutant and its increase in HE patients was attributed to impaired liver catabolism, whereas increased 1-methyl-4-(1-methylethenyl)-benzene levels again in HE patients may have originated from an enhanced aromatase activity due to extensive alcohol abuse that could have been responsible for changes in metabolism. HE patients were also characterised by increased octanal, and a compound tentatively identified as 2, 6-dimethyl-7-octen-2-ol levels, which might have resulted from the P450 induction and catabolism of fatty acids [51]. Compounds such as limonene, dimethyl-sulphide, as well as ketones that were also found in the Section 4.1 studies, were given the same possible origin explanations as those discussed in Section 4.1.1. Higher ethanol levels observed in cirrhotic patients are probably caused by increased shunting volumes through portocaval shunts in the liver, preventing the metabolism of endogenous ethanol [35], whereas diminished acetaldehyde levels that were observed in NAFLD, AFLD, and cirrhotic patients were explained by diminished ethanol oxidation [36]. Interestingly, acetaldehyde levels were increased in NAFLD children; however, they were also attributed to the fact that acetaldehyde is a product of liver ethanol metabolism [55]. Finally, TMA either derives from an impaired liver damaged capacity to transform TMA to TMAO (i.e., physiological oxidation of TMA), or it derives from the degradation of dietary phosphatidylcholine and dietary free choline by the intestinal microflora [21,55]. Figure 2 visualises all these suggested pathways.

### 4.3. Liver Diseases Examined by VOC Measured in Faeces, Bile and Urine

#### 4.3.1. VOCs in Faeces

Raman et al. [60] sampled obese NAFLD presence patients and healthy controls to analyse and compare VOCs patterns in the headspace of faecal matter by running a GC-MS analysis. They found a core group of ester VOCs that was more abundant in obese NAFLD patients than healthy controls (normal liver and lean). This suggests that obese NAFLD patients have altered microbiome composition. Using binary data, they found 12 compounds that were significantly less common and 18 compounds that were more common in the faecal headspace of NAFLD patients than in healthy controls. Ester compounds composed most of the identified VOCs (i.e., aliphatic esters of ethanoic, butanoic, propanoic, and pentanoic acids). Most of these compounds were short-chain aliphatic alcohols and carboxylic acids derivatives. The origin of volatile esters coming from the gut microbiota [60] is still elusive. However, it is believed that bacterial enzymes such as esterases could catalyse reactions by using organic acids and alcohols; thus, leading to the formation of ester VOCs such as those found in their study [60]. Ethanol was seen as a ubiquitous compound since it was present in both NAFLD and healthy individuals; nevertheless, these findings do not allow conclusions to be drawn as they are only qualitative findings. Many confounding factors were present, as the researchers did not account for different diets, environment, or smoking. The study population did not include non-NAFLD obese patients; therefore, it is unknown if VOC characteristics are due to NALFD or obesity. The VOCs detected in the [60] study in the faecal headspace (esters of ethanoic, butanoic, propanoic, and pentanoic acids) belonged to the same classes as the compounds found by papers analysing breath (2-butanone, 2-pentanone, ethane). This suggests that breath VOCs could be derivatives of VOCs created by gastrointestinal bacteria, as argued in [60].

#### 4.3.2. VOCs in Bile

In 2015, Navaneethan et al. [37] published a pilot study in patients with primary sclerosis cholangitis (PSC), a risk factor for cholangiocarcinoma (CCA). Bile samples from the endoscopic bile repository were selected for analysis, of which some were PSC only patients, and some were PSC with CCA patients. Their objective was to identify potential VOCs in the bile headspace to discriminate CCA progression in PSC patients. They ran a SIFT-MS analysis, and they reported the following significantly different compounds: ethanol, acetonitrile, acrylonitrile, 3-methyl-trexane, benzene, carbon disulphide, acetaldehyde, dimethyl-sulphide, and 2-propanol. Combining 3-methyl-hexane, acrylonitrile, and benzene, they built a predictive model to diagnose PSC patients with CCA with a sensitivity and specificity of 90.5% and 72.7%, respectively. Benzene, an environmental pollutant originating from tobacco smoke and vehicle exhaust [37], was found alongside acrylonitrile and acetonitrile to be significantly less abundant in patients with CCA than PSC only patients. Additionally, dimethyl-sulphide, carbon disulphide, and mercaptopurines, which are products of incomplete metabolism in the liver of sulphur-containing amino acids [37], are less prominent in PSC patients with CCA. However, it should be noted that all of the compounds found in the [60] study, except for acetonitrile and acrylonitrile, have also been associated with liver disease by multiple papers analysing breath VOCs [33,47,48,55,66]. The [37] study illustrates that bile VOC analysis has potential for clinical applications. However, bile collection requires invasive procedures, and thus, it may not be the best path towards alternative VOC diagnosis of liver disease.

#### 4.3.3. VOCs in Urine

Navaneethan et al. [61] published another pilot study conducted on urinary samples consisting of patients with CCA, patients with pancreatic cancer, and patients with benign biliary strictures (PSC, chronic pancreatitis, and papillary steatosis). They aimed to diagnose biliary strictures in urinary VOCs by running a SIFT-MS analysis. They found that ethane levels were significantly higher in PSC strictures compared to CCA patients. They also found that 2-propanol and carbon disulphide levels were lower in malignant strictures, which is in line with their previous study in the bile [37]. They generated a model using ethane and octane, which predicted CCA and malignancy with sensitivity and specificity of 80% and 100%, respectively.

Arasaradnam et al. [53] published a proof-of-principle study also focused on urinal VOC analysis. The patients recruited were NASH cirrhotic (NASH-C), NASH non-cirrhotic, and NAFLD; healthy controls (normal liver) were also recruited. Their objective was to determine whether different stages of NAFLD and NASH had specific urinary VOC patterns and to pursue this, they ran a field asymmetric ion mobility spectrometry (FAIMS) analysis. The [53] study revealed that a urinary VOC breath-print could discriminate between all liver disease patients and healthy controls with low sensitivity of 58% and high specificity of 93%, and an AUC of 0.73. Arasaradnam et al. argued that these results suggest that different liver disease conditions create other chemicals [53]. The analysis also showed that urinary VOCs could distinguish between NASH and NAFLD with a sensitivity and specificity of 73% and 79%, respectively. Their urinary VOC patterns also distinguished well NASH-C and NASH without cirrhosis [53]. Their study suggests that urinary VOCs could be a potential noninvasive diagnostic tool for diagnosing NAFLD and the different NASH stages.

Finally, Bannaga et al. [63] published another pilot urinal VOC analysis examining HCC. They sampled HCC and non-HCC patients, and they tried to find biomarkers to separate the two classes—the non-HCC cases consisted of healthy and various NAFLD stage individuals, including those with or without fibrosis. They ran a GC-IMS analysis to separate their classes and a GC-MS analysis to identify HCC-related biomarkers. More specifically, the GC-IMS data separated the HCC patients from the fibrotic patients with an AUC of 0.97 (sensitivity 43% and specificity 95%), the HCC patients from the non-fibrotic patients with an AUC of 0.62 (sensitivity 60% and specificity 74%), and the fibrotic from the non-fibrotic patients with an AUC of 0.63 (sensitivity 29% and specificity 90%). Five compounds were identified as significantly different between the HCC and non-HCC patients (i.e., 4-Methyl-2,4-bis(p-hydroxyphenyl)pent-1-ene (2TMS derivative), 2-butanone, 2-hexanone, 1-ethyl-2-methyl-benzene, and 3-butene-1,2-diol,1-(2-furanyl)-) from the GC-MS dataset. All compounds but 2-butanone were significantly lower in HCC patients. Bicyclo[4.1.0]heptane, 3,7,7-trimethyl-, [1S-(1a,3ß,6a)]- and sulpiride were also significantly lower in HCC patients than in fibrotic patients. Bannaga et al. neither verified nor quantified their compounds; however, they gave plausible explanations as to why they may have found these compounds based on existing literature. For instance, they stated that 2-butanone has been reported in breath-related VOCs in liver diseases (this is in agreement with [22,34,46,47]), 1-ethyl-2-methyl-benzene has been identified as a blood biomarker of HCC, whereas 3-butene-1,2-diol,1-(2-furanyl)- has been associated with lung cancer [63].

## 5. Summary

Figure 2 summarises the VOCs reported in the reviewed studies related to chronic liver diseases and their proposed metabolic pathways.

VOC analysis might greatly benefit liver disease diagnosis and prognosis; however, it is apparent from the literature findings that implementation of the VOC analysis in clinical liver practices is not ready yet for routine applications since much more research is needed. All conducted studies are either proof-of-concept studies or of a small sample size. Furthermore, many of the studies presented here did not perform any internal or external validation of their findings. The correction of possible confounding factors was also not considered, and this might have affected their results. Nevertheless, some key concept can be kept from the present review that may point towards the eventual implementation of the VOC analysis in clinical liver practices. Several VOCs have been found in several studies, and as indicated in Figure 2, they have a solid biological explanation. All the compounds reported here are endogenous compounds except for limonene, which is an exogenous compound. This is probably the most striking observation of the present review because it illustrates the possibilities of a different study approach—exogenous VOC exposure. More specifically, one could expose a cohort at a particular limonene concentration with ingestion, sample their breath or maybe urine after exposure, and measure the difference between the inhaled and exhaled limonene concentration to determine liver function. The same principle could be applied to any other exogenous VOC metabolised by the liver. An exogenous VOC analysis enables for a tailored, controlled exposure to a compound of interest, thus providing a better chance in identifying disease-specific markers. Moreover, an exogenous VOC analysis would also be more robust to background VOCs (e.g., environmental VOCs), which are often one of the major confounding factors in the field. It should be noted, however, that there are weaknesses of such an approach too. An exposure to a specific VOC may require patient preparation, but most importantly, it might be source of a potential allergy. Nonetheless, this approach could potentially help with liver disease diagnosis and prognosis since the exhaled concentration could indicate the level of liver impairment. The authors believe that this could push VOC analysis a step forward towards its clinical implementation in the liver research domain and other clinical settings.

## Figures and Tables

**Table 1:** Evaluation of the papers that were included in the present review. Both score columns are shown (i.e., SANRA Scheme 0), medium (i.e., (5–7]), or high (i.e., (7–10]).

Publication	Means of Analysis	SANRA Scores (Averaged)	SANRA and Added Questions Scores (Averaged)	Quality
Friedman et al. 1994 [42]	Breath	6.5	6.25	Medium
Hiroshi et al. 1978 [43]	Breath	7	5	Low
Letteron et al. 1993 [44]	Breath	9	6.5	Medium
Van den Velde et al. 2008 [33]	Breath	9.5	9.25	High
Dadamio et al. 2012 [45]	Breath	10	8.25	High
Pijls et al. 2016 [46]	Breath	10	8	High
Morisco et al. 2013 [22]	Breath	9	8.25	High
Del Rio et al. 2015 [47]	Breath	9	8	High
Eng et al. 2015 [48]	Breath	9.5	7.25	High
Alkhouri et al. 2015 [49]	Breath	10	7.25	High
De Vincentis et al. 2016 [50]	Breath	9	5.75	Medium
Khalid et al. 2013 [51]	Breath	9	6.75	Medium
O’Hara et al. 2016 [52]	Breath	10	8.5	High
Arasaradnam et al. 2015 [53]	Breath	9	5.5	Medium
Solga et al. 2006 [5]	Breath	9	6.75	Medium
Verdam et al. 2013 [54]	Breath	9	6.25	Medium
Alkhouri et al. 2013 [55]	Breath	9.5	6.75	Medium
Millonig et al. 2010 [35]	Breath	7.5	7.5	High
Hanouneh et al. 2014 [21]	Breath	9	7.75	High
Qin et al. 2010 [56]	Breath	7.5	6	Medium
Sinha et al. 2019 [57]	Breath	10	7	Medium
Ferrandino et al. 2020 [58]	Breath	10	7	Medium
Miller-Atkins et al. [59]	Breath	10	8.75	High
Raman et al. 2013 [60]	Faeces	9	6.75	Medium
Navaneethan et al. 2015 [37]	Bile	9	6.75	Medium
Navaneethan et al. 2015 [61]	Urine	9	6.75	Medium
Arasaradnam et al. 2012 [62]	Urine	8.5	6	Medium
Bannaga et al. 2021 [63]	Urine	9.5	7	Medium

**Table 2:** A summary of the compounds that were found as significant in more than one of the examined research papers in the present review. What is believed to be the biological origin of each compound is described here too.

Compound	Number of Times	Biological Origin
Dimethyl-sulphide	11	Incomplete metabolism of sulphur-containing amino acids in the transamination pathway—cytochrome C oxidase deficiency
Limonene	7	Limonene is not produced in the human body—metabolised by the P450 enzymes CYP2C9 and CYP2C19—accumulates in the fat of patients
Acetone	7	Due to hepatic insulin resistance that leads to an increase in triglycerides, free fatty acids and ketones
Ethanol	7	Due to increased shunting volumes through portocaval shunts
Isoprene	6	A by-product of cholesterol biosynthesis—the intestinal microbiota may generate isoprene too
Acetaldehyde	6	Oxidation product in ethanol metabolism—CYP2E1 is induced
2-Pentanone	5	Due to hepatic insulin resistance—inhibition of CYP2E1
Carbon-disulphide	4	The oxidative metabolism of carbon disulphide—also due to incomplete metabolism of sulphur-containing essential systems
2-Butanone	4	Due to hepatic insulin resistance, formed during lipolysis—inhibition of CYP2E1
Benzene	4	Environmental pollutant
Pentane	3	Lipid peroxidation—a by-product of the cytochrome P450 metabolism
Hydrogen-sulphide	3	Incomplete metabolism of sulphur-containing amino acids in the transamination pathway—cytochrome C oxidase deficiency (less stable than dimethyl-sulphide)
Ethane	3	Lipid peroxidation of polyunsaturated fatty acids—a by-product of the cytochrome P450 metabolism
Trimethyl-amine (TMA)	3	The intestinal microflora degrades dietary phosphatidylcholine to form trimethylamine—trimethylamine is metabolised by the hepatic flavin monooxygenase family of enzymes
2-Nonene	3	It is yet to be discovered—it has been linked to oxidative stress
2-Propanol	2	It is yet to be discovered—it is speculated to be related to inflammatory processes and/or lipid peroxidation
Indole	2	Derived from the catabolism tryptophan
Dimethyl-selenide	2	Excretion product of the essential micronutrient selenium
Methanol	2	Metabolised mainly by alcohol dehydrogenase—pectin degradation—an imbalance of microflora composition in cirrhotic patients
2-Octanone	2	Due to hepatic insulin resistance, formed during lipolysis—inhibition of CYP2E1
Octane	2	Metabolised by the cytochrome P450 enzymes
Alpha-pinene	2	Metabolised by the cytochrome P450 enzymes
Tridecane	2	It is yet to be discovered—it is speculated that it is related to inflammatory processes and/or lipid peroxidation
Styrene	2	Exogenous sources such as industrial materials—it is oxidised by cytochrome P450

**Table 3:** Summary of the papers that examined cirrhosis/CLD patients against healthy cohorts. The arrows show the VOC abundance in the CLD group compared to the healthy group in the study design.

Author/Year	Study Design	Analytical Method	VOCs Identified as Significant	Discriminatory Performance
Friedman et al. 1994	24 cirrhotic CLD vs. 24 healthy	GC-MS	Hydrogen-sulphide ↑ Limonene ↑	Not reported
Van den Velde et al. 2008	52 cirrhotic CLD vs. 50 healthy	GC-MS	Acetone ↑ Dimethyl-sulphide ↑ 2-butanone ↑ 2-pentanone ↑ Indole ↓ Dimethyl-selenide ↓	100% sensitivity 70% specificity
Dadamio et al. 2012	35 cirrhotic CLD vs. 49 healthy	GC-MS	Dimethyl-sulphide ↑ Acetone ↑ 2-butanone ↑ 2-pentanone ↑ Indole ↓ Phenol ↓ Dimethyl-selenide ↓ Isoprene ↑ Ethane ↑ Pentane ↑	83% sensitivity 100% specificity
Morisco et al. 2013	12 cirrhotic CLD vs. 14 healthy	PTR-MS	Heptadienol ↑ Methanol ↑ 2-butanone ↑ 3-pentone ↑ 2-octanone ↑ 2-nonanone ↑ Monoterpene ↑ P-cymene ↑	83% sensitivity 86% specificity
Del Rio et al. 2015	31 cirrhotic CLD vs. 30 healthy	PTR-MS	Methanol ↑ 2-butanone ↑ Carbon-sulphide ↑ 2-pentanone ↑ Limonene ↑	97% sensitivity 70% specificity
Pijls et al. 2016	34 cirrhotic CLD vs. 87 non-cirrhotic CLD	GC-MS	Dimethyl-sulphide ↑ Terpene (limonene) ↑ 2-methyl-butanal ↓ Propanoic acid ↑ Octane ↑ Terpenoid ↑ 3-carene ↑ 1-hexadecanol ↓ C16H34 ↓	83% sensitivity 87% specificity
De Vincentis et al. 2016	65 cirrhotic CLD vs. 39 non-cirrhotic CLD	E-nose	Not available	86.2% sensitivity 98.2% specificity
Eng et al. 2015	49 cirrhotic CLD children vs. 55 healthy children	SIFT-MS	1-decene ↑ 1-heptene ↑ 1-octene ↑ 3-methyl-hexane ↑ 1-nonene ↓ (E)-2-nonene ↓ Dimethyl-sulphide ↓	0.97 AUC

**Table 4:** Summary of the papers that examined cirrhotic, non-cirrhotic and various pre-cirrhotic stage occasion patients against each other. The arrows show (if applicable) whether a VOC level increased or decreased in the first group compared to the second group in the study design.

Author/Year	Study Design	Analytical Method	VOCs Identified as Significant	Discriminatory Performance
Alkhouri et al. 2015	20 advanced fibrosis vs. 41 mild fibrosis	SIFT-MS	Acetone ↓ Benzene ↓ Carbon disulphide ↓ Isoprene ↓ Pentane ↓ Ethane ↓	0.85 AUC *(Isoprene model)*
Khalid et al. 2013	11 alcoholic cirrhotic with HE vs. 23 alcoholic cirrhotic without HE	GC-MS	Methyl-vinyl ketone ↓ Isothiocyanato-cyclohexane ↑	90% sensitivity 87% specificity
	34 alcoholic cirrhotic vs. 13 non-alcoholic cirrhotic		Undecane ↑ Unknown ↓	78.3% sensitivity 69.2% specificity
	34 alcoholic cirrhotic vs. 7 harmful drinkers		1-methyl-4-(1-methyl-ethenyl)-benzene ↑ Unknown ↓ Unknown ↓	88% sensitivity 85% specificity
	7 harmful drinkers vs. 15 healthy		Octanal 2,6-dimethyl-7-octen-2-ol Unknown	71% sensitivity 93% specificity
	13 non-alcoholic cirrhotic vs. 15 healthy		Methyl-vinyl ketone 1-methyl-2-(1-methyl-ethyl)-benzene (o-cymene) Unknown	92% sensitivity 100% specificity
	34 alcoholic cirrhotic vs. 15 healthy		Heptane 1-methyl-2-(1-methyl-ethyl)-benzene Phellandrene 2-methyl-hexane	97% sensitivity 93% specificity
O’Hara et al. 2016	11 cirrhotic HE vs. 11 cirrhotic without HE vs. 7 history of HE vs. 30 healthy	PTR-MS	Limonene ↑	Not reported
	10 without HCC vs. 21 HCC vs. 30 healthy		Limonene ↑	Not reported
Qin et al. 2010	30 HCC vs. 36 healthy	GC-MS-SPME	3-hydroxy-2-butanone ↑ Styrene ↑ Decane ↑	83.3% sensitivity 91.7% specificity
	30 HCC vs. 27 cirrhotic without HCC		3-hydroxy-2-butanone ↑ Styrene ↑	70% sensitivity 70.4% specificity
Ferrandino et al. 2020	32 cirrhotic without HCC vs. 12 cirrhotic with HCC vs. 40 healthy controls	GC-MS	Limonene ↑	73% sensitivity 77% specificity
Miller-Atkins et al. 2020 † only the three most significant metabolite associations for each disease comparison are shown in the column of significant compounds	112 non-cirrhotic HCC vs. 54 healthy	SIFT-MS	(E)-2-nonene ↑ Ethane ↑ Benzene ↑ Hydrogen sulphide ↓	*Healthy* vs. *all the rest* 76% sensitivity 97% specificity
	30 cirrhotic without HCC vs. 54 healthy		Trimethyl-amine ↓ Propanol ↓	*Cirrhotic* vs. *all the rest* 40% sensitivity 96% specificity
	49 PH vs. 54 healthy		(E)-2-nonene ↑ Acetaldehyde ↑ Ethane ↑ Hydrogen sulphide ↓	*HCC* vs. *all the rest* 73% sensitivity 71% specificity
	51 CRLM vs. 54 healthy		(E)-2-nonene ↑ Acetaldehyde ↑ Triethyl-amine ↑ Acetone ↓	*CRLM* vs. *all the rest* 51% sensitivity 94% specificity
	112 non-cirrhotic HCC vs. 30 cirrhotic		Acetone ↓ Acetaldehyde ↓ Dimethyl-sulphide ↓ Ethanol ↑	*PH* vs. *all the rest* 58% sensitivity 93% specificity
Arasaradnam et al. 2016	22 non-cirrhotic HE vs. 20 healthy	E-nose	Not available	88% sensitivity 68% specificity
	13 covert non-cirrhotic HE vs. 9 overt non-cirrhotic HE		Not available	79% sensitivity 50% specificity
Solga et al. 2008	16 moderate to severe steatosis vs. 11 less steatosis	GC	Ethanol ↑ Acetone ↑	Not reported
	24 NASH vs. 24 without NASH		Acetone ↑	Not reported
Verdam et al. 2013	39 NASH vs. 26 without NASH	GC-MS	n-tridecane ↑ 3-methyl-butanonitrile ↑ 1-propanol ↑	90% sensitivity 69% specificity
Alkhouri et al. 2013	37 obese NAFLD vs. 23 obese without NAFLD	SIFT-MS	Isoprene ↑ Acetone ↑ Trimethylamine ↑ Acetaldehyde ↑ Pentane ↑	0.76 AUC
Millonig et al. 2010	37 cirrhotic vs. 35 healthy	IMR-MS	Ethanol ↑	0.88 AUC
	91 liver diseased vs. healthy		Acetaldehyde ↑ Ethanol ↑ Isoprene ↑	0.94 AUC
	34 NAFLD vs. healthy controls		Acetaldehyde ↑	0.96 AUC
	20 AFLD vs. 35 healthy		Acetaldehyde ↑ Isoprene ↑	0.97 AUC
	20 AFLD vs. 34 NAFLD		Isoprene ↑	0.95 AUC
Letteron et al. 1993	89 alcohol abusers vs. 52 liver diseased vs. 42 healthy	GC-FID	Ethane ↑	Not reported
Hanouneh et al. 2014	80 liver diseased vs. 43 healthy	SIFT-MS	2-propanol ↑ Acetaldehyde ↑ Acetone ↑ Ethanol ↑ Pentane ↑ Trimethylamine ↑	Not reported
	40 cirrhotic AH vs. 40 cirrhotic AD		Acetaldehyde ↑ Acetone ↑ Pentane ↑ Trimethylamine ↑	97% sensitivity 72% specificity (Acetone-pentane-trimethylamine)

## Supplementary Materials

The following are available online at https://www.mdpi.com/article/10.3390/metabo11090618/s1, Table S1: Liver Impairment—The Potential Application of Volatile Organic Compounds in Hepatology.
