# Supplementary material for: Liver Impairment—The Potential Application of Volatile Organic Compounds in Hepatology
Source: Metabolites. 2021 Sep 11;11(9):618. doi: 10.3390/metabo11090618 (PMC8471934; doi:10.3390/metabo11090618)
Supplement: Supplementary file 1 [file metabolites-11-00618-s001.zip › metabolites-1380162-supplementary.pdf]

Supplementary materials

Table S1: Added questions to further and more strictly evaluate the papers included in the present review.

Added questions
1) Did the authors correct or control for confounding factors such as diet, smoking, alcohol, medication, and place/time of sample collection?<br>2) Was the sample size sufficient?<br>3) Did the authors perform any statistical analysis? If so, did they correct for multiple testing? Also, did the authors validate their results? If so, did they perform internal (within the same cohort) or even external (new independent cohort) validation or both?<br>4) Where the study classes balanced?<br>5) Did the authors give the biological or non-biological origin of the compounds they found significant in their study?

Table S2: Added question comments as to why each publication was given its respective quality value. If no explanation is given, it means that all issues raised in the added questions were addressed by that particular study.

Publication	Quality	Explanation
Friedman et al. 1994 [48]	Medium	They did not mitigate for confounding factors; small sample size; they did not establish the origin of the found compounds; no statistical modelling was performed
Hiroshi et al. 1978 [46]	Low	They did not mitigate for confounding factors; small sample size; they did not establish the origin of the found compounds; no statistical modelling was performed
Letteron et al. 1993 [69]	Medium	They did not validate their results in terms of statistical modelling; imbalanced study classes; they did not establish the origin of the found compounds
Van den Velde et al. 2008 [34]	High	-
Dadamio et al. 2012 [49]	High	-
Pijls et al. 2016 [51]	High	-
Morisco et al. 2013 [22]	High	They did not validate their results in terms of statistical modelling
Del Rio et al. 2015 [50]	High	They did not validate their results in terms of statistical modelling
Eng et al. 2015 [56]	High	They did not validate their results in terms of statistical modelling
Alkhouri et al. 2015 [60]	High	They did not mitigate for confounding factors; imbalanced study classes
De Vincentis et al. 2016 [52]	Medium	They did not validate their results in terms of statistical modelling; no compounds were identified (due to the e-nose technology itself)
Khalid et al. 2013 [61]	Medium	They did not mitigate for confounding factors; imbalanced study classes
O'Hara et al. 2016 [45]	High	They did not validate their results in terms of statistical modelling
Arasaradnam et al. 2015 [74]	Medium	Small sample size; imbalanced study classes; they did not establish the origin of the found compounds
Solga et al. 2006 [5]	Medium	They did not mitigate for confounding factors; they did not validate their results in terms of statistical modelling
Verdam et al. 2013 [66]	Medium	They did not mitigate for confounding factors; they did not validate their results in terms of statistical modelling
Alkhouri et al. 2013 [67]	Medium	They did not mitigate for confounding factors
Millonig et al. 2010 [36]	High	-

Hanouneh et al. 2014 [21]	High	-
Qin et al. 2010 [62]	Medium	They did not validate their results in terms of statistical modelling; imbalanced study classes
Sinha et al. 2019 [68]	Medium	Small sample size
Ferrandino et al. 2020 [63]	Medium	They did not mitigate for confounding factors
Miller-Atkins et al. [64]	High	-
Raman et al. 2013 [72]	Medium	They did not mitigate for confounding factors; they did not validate their results in terms of statistical modelling
Navaneethan et al. 2015 [38]	Medium	Small sample size; they did not validate their results in terms of statistical modelling
Navaneethan et al. 2015 [73]	Medium	They did not validate their results in terms of statistical modelling
Arasaradnam et al. 2012 [65]	Medium	They did not mitigate for confounding factors; small sample size; no compounds were identified (due to the e-nose technology itself)
Bannaga et al. 2021 [75]	Medium	They did not mitigate for confounding factors; small sample size
